# Does Working-Memory Training Given to Reception-Class Children Improve the Speech of Children at Risk of Fluency Difficulty?

**DOI:** 10.3389/fpsyg.2020.568867

**Published:** 2020-11-17

**Authors:** Peter Howell, Li Ying Chua, Kaho Yoshikawa, Hannah Hau Shuen Tang, Taniya Welmillage, John Harris, Kevin Tang

**Affiliations:** ^1^Division of Psychology and Language Sciences, Experimental Psychology, University College London, London, United Kingdom; ^2^Division of Psychology and Language Sciences, Linguistics, University College London, London, United Kingdom; ^3^Department of Linguistics, University of Florida, Gainesville, FL, United States

**Keywords:** fluency difficulty, word-finding difficulty, working memory, English as an additional language, developmental stuttering disorders, diversity

## Abstract

Procedures were designed to test for the effects of working-memory training on children at risk of fluency difficulty that apply to English and to many of the languages spoken by children with English as an Additional Language (EAL) in UK schools. Working-memory training should: (1) improve speech fluency in high-risk children; (2) enhance non-word repetition (NWR) (phonological) skills for all children; (3) not affect word-finding abilities. Children starting general education (*N* = 232) were screened to identify those at risk of fluency difficulty. Children were selected who were at high-risk (12), or low-risk (27) of fluency difficulty. For the low-risk children 10 received, and 17 did not receive, the working-memory training. All children in the treatment groups received working-memory training over a 2-week period. For the high-risk group, fluency improved and lasted for at least a week after the end of the study. Phonological skills improved in this group and in the low-risk group who received the training and the improvements continued for at least a week. The low-risk group who did not receive working-memory training showed no improvements, and no group improved word-finding ability.

## Introduction

It is widely agreed that children who have speech and language communication needs (SLCN) should receive attention as early as possible (Bercow, 2008). This article examined the effects of working-memory (WM) training for school-entry age children who have one form of SLCN (fluency difficulty). As background, the way children with fluency difficulty can be identified, issues associated with testing in schools, and how to provide information about children in useful ways for schools, and for Speech Language Pathologists (SLPs), to use are reviewed.

### Identification of Children With Fluency Difficulty in Schools

Early identification of fluency difficulty is essential (Yairi and Ambrose, 2005; Bercow, 2008). Almost all children in the UK attend reception classes (start of general education at around age five years) and this offers the opportunity to examine them so that any who have fluency difficulty are identified (Howell, 2013; Howell et al., 2017a). Pediatric fluency problems such as stuttering have usually started by this age but have not persisted for so long that they become resistant to remediation (Howell, 2010a). Certain approaches that would allow children with fluency difficulty to be identified in schools are ruled out. For example, assessment of all school children by SLPs would be expensive and would not be appropriate since the majority of the children are fluent. In addition, full clinical assessments cannot be conducted in schools because it is sometimes difficult to collect family history and other information for several reasons such as poor response rate or when a child only has one parent.

Howell et al.’s (2017a) procedure for identifying children with fluency difficulty can be used for work in schools. It employs a spontaneous speech sample to estimate the incidence of fragmentary symptoms (part-word repetition, prolongation or word breaks). The percentage occurrence of these symptoms out of all syllables spoken (%SS) is calculated (Riley, 2009). Children whose scores are above a threshold %SS are designated as having fluency difficulty. This procedure is partly based on Riley’s (2009) instrument that combines %SS, the duration of the three longest stutters and a measure of physical concomitants as an index of stuttering severity. The components Riley included in addition to %SS are not necessary as Mirawdeli and Howell (2016) showed that accuracy in identifying children with fluency difficulty was superior if %SS alone was used.

Riley’s (2009) SS symptoms were developed for assessing stuttering, but other fluency problems can occur in children attending mainstream schools. It was thought that these other SLCN had distinctive symptoms that would improve identification of the wider class of pediatric fluency difficulties, but Campbell (2014) showed that this was not the case. She showed that Riley’s symptoms classified children as fluent or as having fluency difficulty better than schemes that included additional fluency symptoms. Hence, there does not seem to be any compelling reason to modify Riley’s symptom set when it is used to identify children with fluency difficulty in schools.

Word-finding difficulty (WFD) occurs when children do not know, or cannot retrieve, a subsequent word (German, 1991). In these cases, hesitancy in speech reflects vocabulary deficiencies so procedures for improving fluency difficulty would not be effective with these children. Howell et al. (2017a) used one of Campbell’s (2014) hesitancy symptoms (whole-word repetitions, WWR) to identify children with WFD and to distinguish them from those who have fluency difficulty. In the study reported in this paper, children who only had high rates of %WWR were designated as having WFD (Howell et al., 2017a), not fluency difficulty. This procedure uses %WWR and %SS separately in analyses but does not imply that WWR are absent when children have fluency difficulty. However, it does require that for children to be considered as having fluency difficulty, they have to exhibit SS as well as (optionally) WWR. The approach of not using WWR for assessing fluency difficulty is consistent with Riley (2009).

This section has shown that Howell et al.’s (2017a) procedure using symptoms derived from Riley (2009) is suitable for identifying children with fluency difficulty in schools. Campbell’s (2014) analyses showed that Riley’s (2009) %SS is the optimum symptom set to use with samples of children with different types of fluency difficulty. Children who only show WWR would not be expected to improve after WM training as their difficulty stems from word-finding problems.

### Testing in Schools

Once fluency difficulty has been identified, children should be referred to SLPs as soon as possible since children experience several challenges if the issues are not addressed (Antoniazzi et al., 2010; Snow, 2014). SLPs have limited time to visit schools (Mirawdeli, 2016), which restricts the service that they can provide. One way to extend provision would be for SLPs to instruct teachers on how to identify children with fluency difficulty (Dockrell and Lindsay, 2001). This approach is consistent with the recent shift toward a consultative model of service delivery in which SLPs advise educational staff on how to support children with different needs (Law et al., 2002). The procedure examined here was designed so that it conforms to this criterion and can be administered in schools.

### Coordination of School and SLP Services

Different professional groups make decisions on which children have fluency difficulty in different ways (Bercow, 2008): Schools are concerned when fluency difficulty affects education, whereas, SLPs consider wider issues associated with communication in various social, and work, settings (Dockrell et al., 2017). Consequently, since schools are in control of budgets, decisions about children’s needs could be pre-empted and result in some children not being referred to SLPs even though this would be useful.

Concerns about coordination of actions between schools and SLPs are ameliorated by ensuring that any procedure carried out in schools does not conflict with anything an SLP may subsequently do with these children. One way of achieving this is to design procedures that have positive effects on all children (not just those with fluency difficulty). Specifically, procedures for training WM in schools would be appropriate as they are innocuous to children who are falsely diagnosed with difficulty and given WM training as the training should benefit children with or without fluency difficulty.

Failure to identify children who have fluency difficulty is another potential problem when procedures are conducted in schools (Howell, 2010a). No-one knows how many children who would benefit from referral to SLPs are missed since there is no standard way of assessing all children for fluency difficulty. However, this issue is mitigated to some extent by using a validated procedure for identifying children with fluency difficulty (Howell et al., 2017a).

### Design Considerations for an In-School Procedure for Fluency Difficulty

A suitable procedure has to apply to a wide range of pediatric speech issues, and has to be appropriate for children who speak English alone or use English as an Additional Language (EAL) when they start school. Also, the procedure should not conflict with SLP practices that might subsequently be required with any of the children. WM training is a possibility as it supports performance on cognitive activities such as speaking (Baddeley, 1981) and people who have fluency problems have WM deficits (Daneman, 1991). The phonological loop aspect of WM helps retain representations by rehearsing them (Baddeley and Hitch, 1974) and its quality can be assessed by non-word repetition (NWR) performance (Gathercole et al., 1994). WM training would benefit all children including those who are fluent. Thus, in typical participants, NWR performance correlates well with conventional measures of phonological memory such as auditory digit span (Gathercole et al., 1994; Gray, 2003). WM is adversely affected (i.e., NWR performance is poor) in many types of SLCN. Examples include developmental language delay, formerly called Specific Language Impairment (Gathercole and Baddeley, 1990), dyslexia (Catts et al., 2005), Autistic Spectrum Disorder (Whitehouse et al., 2008), and stuttering (Bakhtiar et al., 2007). Although there is firm evidence that WM and fluency difficulty are related, WM does not explain how fluency difficulties arise, since it is not known what causes WM to vary across individuals. Even though this information is lacking, providing that children with fluency difficulty have a WM deficit, WM training can be legitimately offered to them.

### The Present Study

Children were assessed to identify which of them should be given a WM procedure for fluency difficulty. Howell et al.’s (2017a) screening procedure uses fragmentary symptom (SS) counts from samples of English spontaneous speech in order to distinguish children at high-risk of fluency difficulty from the remaining (low-risk) children. Consequently, children with symptoms of WFD alone (e.g., frequent WWR and pausing in their speech) are not regarded as having fluency difficulty. This identification procedure distinguishes children with fluency difficulty from those with WFD and, using the UNWR procedures described below, is applicable whether English is a child’s first or additional language.

Comblain’s (1994) WM training procedure successfully trains rehearsal strategies in children with Down syndrome and it was adapted for the present study. The goals were to improve verbal WM skills and to establish whether this affected speech fluency in children at high-risk of fluency difficulty (high-risk with WM training). The procedure was also given to a group of children at low-risk of fluency difficulty as it should improve their WM (low-risk with WM training) and a second low-risk group who did not receive any training (low-risk no WM training). Children with WFD would not benefit from the WM procedure since it does not address vocabulary issues.

Measures of fluency difficulty (%SS), WFD (%WWR) and NWR performance (scores on Howell et al.’s 2017a, UNWR test) were obtained at three phases (pre-training, post-training and a week after the end of training) for the high-risk and low-risk groups who received WM training and at equivalent times for the low-risk no WM training group. The low-risk no WM training group performed another, non-WM, task in the training period. UNWR is a NWR test that provides a measure of phonological-loop rehearsal. It can be applied with a wide range of the languages spoken in UK schools so that performance across children who use these languages can be made. The fluency and WFD analyses are also appropriate for children with EAL (Howell et al., 2017a). Performance measures for the three groups were assessed for changes across phases: pre to post comparisons show effects of the training; pre to follow-up and post to follow-up changes both allow retention to be determined.

In summary, the WM training was evaluated on three measures: fluency (%SS); WFD (%WWR); and phonological ability (UNWR score). Each measure was obtained at three phases; pre and post training and after a 1-week follow-up. Three groups of children were tested: one with high-risk of fluency difficulty who received the WM training; two low-risk groups one of whom also received the WM training and one who performed a related activity that did not involve WM. It was predicted that: (1) only the high-risk group would improve their fluency across the phases; (2) measures of WFD would not be affected by the training for any of the groups; (3) phonological ability would improve for both groups who received the WM training (low-risk and high-risk) but not for the low-risk no WM training group.

## Materials and Methods

### Participants Screened

All 232 reception class children from five primary schools were assessed. The schools were in Ipswich (one) and the London boroughs of Hackney (one) and Merton (three). Ipswich’s population was 133,400 in 2014, of which 82.9% were White British and the average weekly pay for men was £456^1^, which was lower than that of England overall (£513). Hackney’s population was 273,526 in 2016 of which 36.2% were White British and average weekly pay for all workers was £613.30^2^. Merton’s population was 199,700 in 2015, 75.0% of residents were white British and median gross weekly pay was £535.50^3^.

Potential candidates for the training study were selected from the cohort of 232 children. Children who had EAL were included provided that their first language was one to which the UNWR applies^4^ (31 children with other additional languages were excluded). Also excluded were seven bilingual children who spoke English and another language fluently, and two children who had diagnosed hearing loss. This left 192 children of whom 103 were female (38 had EAL) and 89 were male (22 had EAL). The overall mean age was *M* = 4.55 with standard deviation *SD* = 0.52. The gender groups did not differ statistically in age by Wilcoxon rank sum test with continuity correction *U* = 4785.5, *p* = 0.60. The languages the children spoke were: English (68.75%); Urdu-Hindi (6.25%); Polish (5.73%); Bengali (5.73%); European Portuguese (4.17%); Romanian (2.08%); German (1.56%); Bulgarian (1.04%); Turkish (1.04%); Swedish (1.04%); Latvian (0.52%); Czech (0.52%); Russian (0.52%); Pashto (0.52%), and Dutch (0.52%). A breakdown of the 192 children by is given in Table 1. The study had institutional ethical approval (0078/004) and informed consent was obtained from schools and parents.

**TABLE 1:** Participant details for 192 children assessed broken down by language.

Language	Females	Males	Mean Age ± *SD*
English	65	67	4.51 ± 0.53
Urdu-Hindi	6	6	4.58 ± 0.50
Polish	10	1	4.63 ± 0.29
Bengali	5	6	4.57 ± 0.22
European Portuguese	4	4	4.67 ± 0.65
Romanian	4	0	4.57 ± 0.37
German	1	2	3.97 ± 0.51
Bulgarian	1	1	4.64 ± 0.05
Turkish	1	1	5.46 ± 0.13
Swedish	1	1	5.08 ± 0
Latvian	1	0	5.13 ± 0
Czech	1	0	5.11 ± 0
Russian	1	0	4.80 ± 0
Pashto	1	0	5.18 ± 0
Dutch	1	0	5.68 ± 0

**Number of participants for each gender and overall mean age and standard deviation are given.**

### Selection of Participants for the WM Training Groups

Speech samples from the 192 children were analyzed to determine who should be included in the groups employed in the training study. A 10–15 min speech sample was obtained in a quiet room using a Sennheiser SC 660 USB ML headset connected to a laptop. There were apparatus problems for 10 children and recordings were made using the internal microphone of a Sony Vaio Pro 13 Laptop instead. Picture material from Riley (2009) was used to elicit speech samples.

%SS and %WWR were obtained from the spontaneous speech samples. The total number of syllables in each sample was calculated (Riley, 2009). To obtain %SS, the number of SS (part-word repetitions, prolongations and word breaks) was obtained and expressed as a percentage out of all syllables spoken (Riley, 2009). For %WWR, multiple iterations of single whole words were counted as a single event. The total syllable count for %WWR was adjusted by subtracting the number of syllables in the repeated units from the total syllable count used in the %SS analyses. For example, the WWR “whether whether whether” has two repeated units (underlined) containing four syllables in total that would be subtracted from the syllable count. Then %WWR was expressed as their percentage out of all syllables. This made the %WWR estimates equivalent to the %SS of Riley (2009) apart from the types of events included.

Inclusion criteria for high-risk of fluency difficulty were (a) a combined %SS and %WWR scores of greater than 3% (Yairi and Ambrose, 2005); and/or (b) they displayed articulation difficulties such as reluctance to utter anything more than isolated monosyllables, as noted by teachers and confirmed by experimenters^5^. Twelve children were identified and received the training. Six were female (four had EAL) and six were male (zero had EAL). The mean age of the high-risk with WM training group was 4.55, with *SD* = 0.36. The inclusion criteria for children in the low-risk groups were: (1) a %SS below 1%; and (2) their native language matched with one used by the children in the high-risk WM training group. In the low-risk with WM training group, there were five females (two had EAL) and five males (one EAL) with *M* age 5.38, and *SD* = 0.24. In the low-risk no WM training group there were eight females (six had EAL) and nine males (one EAL) and *M* age was 4.43, with *SD* = 0.40. Table 2 summarizes the dependent variables and the predictors for the three groups of participants.

**TABLE 2:** Descriptive statistics of the variables (both dependent variables and the predictors) for the high-risk with WM training, low-risk no WM training and low-risk with WM training groups.

	High risk with training			Low risk no training			Low risk with training		
	Mean	*SD*	IQR	Mean	*SD*	IQR	Mean	*SD*	IQR
%SS (Pre)	1.080	0.522	0.730	0.365	0.270	0.382	0.297	0.177	0.115
%SS (Post)	0.501	0.366	0.531	0.209	0.250	0.359	0.103	0.223	0.000
%SS (Follow-up)	0.590	0.773	0.403	0.275	0.225	0.216	0.253	0.360	0.524
WWR (Pre)	0.661	0.855	0.883	0.541	0.431	0.483	1.737	1.289	1.922
WWR (Post)	0.750	0.947	0.605	0.382	0.317	0.324	1.967	1.480	1.792
WWR (Follow-up)	0.884	1.221	1.069	0.439	0.361	0.385	1.622	0.926	1.696
UNWR (Pre)	6.583	3.118	3.000	9.471	4.474	7.000	10.500	3.866	6.750
UNWR (Post)	8.167	2.855	3.000	8.765	4.452	5.000	12.100	4.040	6.750
UNWR (Follow-up)	10.750	4.413	7.250	8.882	4.729	5.000	14.400	3.806	2.750
Age (month)	54.167	4.387	6.250	52.588	4.848	4.000	64.200	2.974	3.750
Gender	M:6, F:6			M:9, F:8			M:5, F:5		
Language group	Monolingual English: 8, EAL: 4			Monolingual English: 10, EAL: 7			Monolingual English: 7, EAL: 3		
School	Hatfeild: 1, Priory: 4, St. Helen’s: 4, Stanford: 3			Hatfeild: 5, Priory: 6, St. Helen’s: 5, Stanford: 1			London Fields: 10		

**The table show the mean, standard deviation and interquartile range for the continuous variables and count information for the categorical variables.**

There were no significant differences in gender across the high-risk and the two low-risk groups nor between the two low-risk groups using χ^2^. There was no significant difference in age between the high-risk with WM training and the low-risk no WM training groups under the Wilcoxon Rank Sum Test (*p* = 0.8106). However, the mean age of the low-risk with WM training group was significantly higher than: (1) the high-risk with WM training group; and (2) the low-risk no WM training group (*p* < 0.001 in both cases). Consequently, corrections for age differences were made in the analyses.

### Measurements at Assessment Phases

Samples of speech were obtained pre, and post training (training lasted 2 weeks) and at follow-up 1 week post training. These were analyzed for %SS and %WWR as described above. In addition, UNWR was used to score NWR performance (Howell et al., 2017a). Recordings of a male phonetician using Southern Standard British English pronunciation with English stress patterns were used for UNWR stimuli (Howell et al., 2017a). These were played to children at their most comfortable volume level and the children repeated the “made-up” words that they heard. There was no time pressure to respond. Each test began with two-syllable long UNWR stimuli and syllable length was increased successively up to five syllables (maximum). There were two practice, and 10 test, trials (randomized) per syllable length. Accuracy was determined on consonants alone immediately after each non-word was produced (Conti-Ramsden et al., 2001) and the correct/incorrect designation was entered manually into the laptop. All ten test stimuli at a given syllable length were delivered but a child only progressed to the next syllable length if eight out of the 10 non-word test stimuli at the current syllable length were correct (significant by Sign test *p* < 0.05). The UNWR score was the total non-words produced correctly.

### Training Materials

Ninety-two colored pictures were selected from the Bank of Standardized Stimuli (BOSS) (Brodeur et al., 2010, 2014). They were high frequency objects with monosyllabic names and the images were printed on 6.5 cm^2^ cards. Familiarity, visual complexity and word frequency ratings were *M* = 4.42, *SD* = 0.32, *M* = 2.41, *SD* = 0.48 and *M* = 4.65, *SD* = 0.87, respectively (Brodeur et al., 2010, 2014). The scales for familiarity and complexity were five point and the word frequency scale was seven points with smaller numbers indicating low scores for the variables.

### Training Procedure

Children who received the WM training (high-risk and low-risk) worked in fixed pairs and memorized a series of items and recalled them in reverse order to that at presentation. There were two components in the training: visual presentation of pictures; and verbal presentation of words (Comblain, 1994).

#### Visual Presentation

The test started with a practice session in which the number of pictures shown gradually increased from one to three. Two trials were given at each number of pictures, hence, 12 of the 92 pictures were required in total [(2 × 1 picture) + (2 × 2 pictures) + (2 × 3 pictures)]. The experimenter first named and placed each picture face down on a table. The children then recalled the pictures in reverse order (e.g., the third, second and then first picture for a trial involving three pictures).

They then received eight two-picture, followed by eight three-picture, test trials. The order of pictures was randomized across pairs of children. Children took turns to respond with different selections of pictures (sampled without replacement) until all 80 pictures had been tested (five trials per child). On each trial one child recalled the pictures whilst the other child paid attention in case they needed to help their partner. Thus, both children in a pair were exposed and attended to the same set of stimuli twice. Children earned a point for each correct trial.

#### Verbal Presentation

The procedure for verbal presentation was the same as during visual presentation with the following exceptions: Pilot work showed that instructing children to report words in reverse order was not successful. Therefore, finger prompts were used to indicate the order of items at presentation and recall. The experimenter raised a finger each time a word was spoken, and pointed to the corresponding finger to signal order of report. For example, a three-word presentation could involve the experimenter saying “dog” *(raises first finger)*, “chair” *(raises second finger)*, “leg” *(raises third finger*). At recall, the experimenter would point in turn to the third, second and first finger and the child should respond leg, chair and dog. The practice stimuli were the same during the second session but the test stimuli differed.

At the end of each of the two presentation formats, all pairs of children had been exposed to all 80 test pictures and 80 verbal items once each during the test trials and once as observer. The total time for the intervention (instructions, practice, and test) was approximately 3 h delivered over 2 weeks.

The low-risk no WM training group performed a non-WM activity with the same visual and verbal materials that were used with the WM training groups. Here children named the items in the order that they were presented by the experimenter. Hence, the non-WM activity involved use of long-term lexical knowledge and short-term memory (storage component alone), but not WM (simultaneous storage and processing).

Two of the authors delivered the training (LYC and HT) to separate groups of children. Training fidelity complied with NIH Behavior Change Consortium recommendations in four areas: (1) *Study design*. Theoretical bases for distinguishing children with fluency difficulty, WFD or typical speech were based on Howell et al. (2017a), the logic outlined in the introduction called for a training procedure that affected speech and cognitive functions favorably and Comblain’s (1994) WM procedure provided that; (2) *Training provision*. PH and LYC developed a written training protocol and PH trained both experimenters who delivered the training using this; (3) *Delivery of training*. Ethics requirements ensured that training was delivered as intended and that any problems were referred to PH (none were reported). Self-monitoring for adherence to procedures was emphasized to LYC and HT during training; (4) *Receipt of training*. The experimenters had worked extensively assessing the children before, after the training and at follow-up including monitoring their ability to use skills and engage in tasks. PH constantly checked with the experimenters about participant engagement. Schools also checked delivery and receipt of the training and verified that this was appropriate (no problems were reported again).

### Reliability

The original judge re-assessed 10 speech samples from screening and 10 speech samples from training for %SS and %WWR. An independent judge also assessed these samples. Intra-judge agreements were lower (mean = 75.55%) than inter-judge agreements (mean = 81.05%) and ranged from 72.3% (intra-judge training for %WWR) to 83.8% (inter-judge training for %WWR).

### Statistical Analyses

Sum coding and backward difference models were fitted in paired-group and individual group analyses using *lme4* (Bates et al., 2015). Sum coding compares the mean of the dependent variable of a specific level to the grand mean of the variable. Backward difference coding compares the mean of the dependent variable of a specific level to the mean of the prior adjacent level. “Paired-group” refers to the selection of two of the three groups whereas “individual group” refers to selection of one of the groups alone. The paired-group analyses were conducted first (three separate analyses) where the pairs were high-risk with WM training versus low-risk with WM training, high-risk with WM training versus low-risk no WM training and low-risk with WM training versus low-risk no WM training. Group and phase (pre, post and follow-up) were factors used to test predictions. Separate analyses were conducted for each of the three dependent variables (%SS, %WWR, UNWR). Hence nine sets of paired-group analyses were conducted (three pairs of groups x three dependent variables). Two contrasts were set up for the phase effects in the sum coding models, (1 = pre to post and 2 = pre to follow-up), and two contrasts were set up for the group x phase effects (phase contrast 1 × test group and phase contrast 2 × test group).

Individual group analyses were only conducted when the overall phase x test group interaction was significant in the above models for ease of interpretation of the effect of phase. For the individual group analyses, sum coding and backward difference analyses were conducted. Sum codings were as above. The backward difference models looked at phase effects across post and follow-up that were not examined in the sum coding models.

All analysis models for the paired-group analyses had three fixed effects of interest (group, assessment phase, and their interaction), four additional fixed effects to control for participants’ demographics (school, gender, age in months, language group) and two random intercepts (participant and training pairing). Age was *z*-score normalized, and the categorical variables (school, gender, and language group) were sum coded to improve the interpretability of the regression coefficients and the collinearity of variables, and to avoid model convergence issues (Wissmann and Toutenburg, 2007; Jaeger, 2009a,b). Individual group analysis models had one fixed effect of interest (assessment phase), the same additional fixed effects of the demographics and the same two random intercepts^6^. The random effects estimated variations that could potentially bias the fixed effect results whilst the fixed effects of the four demographic factors were included to keep the fixed effects of interest conservative. Outliers with residuals more than 2.5 *SD* from the mean were removed after the initial model was built; and the model was refitted using the remaining data to improve the normality of the residuals and to ensure model fits were appropriate (model criticism).

## Results

### Paired-Group Analyses

Changes in %SS, %WWR and UNWR scores for the three pairs of groups over phases (pre, post and at follow-up) were examined. The pairs compared were: (1) high-risk with WM training versus low-risk no WM training to assess whether the performance of the high-risk group became like that of a low-risk group who were not subject to the training; (2) high-risk with WM training versus low-risk with WM training to assess whether the training affected the high-risk group more than the low-risk group; and (3) low-risk with WM training versus low-risk no WM training to assess whether the training affected low-risk groups at all.

### Comparison of High-Risk With WM Training Against Low-Risk No WM Training Groups

**FIGURE 1:**
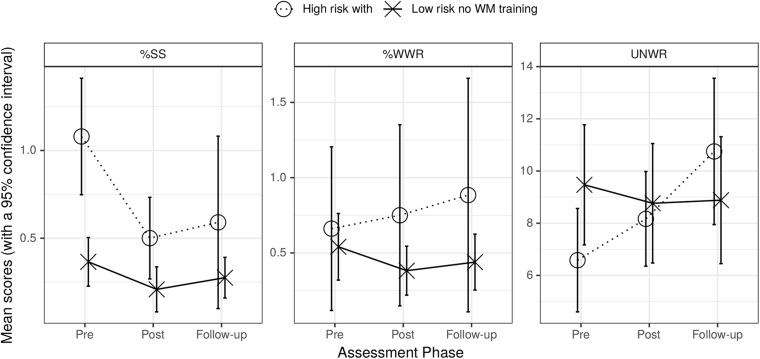
Graph of mean %SS (left), mean %WWR (center), and UNWR scores (right) for high-risk with training and low-risk no WM training groups across assessment phases. Bars are standard errors.

The left section of Figure 1 shows that %SS decreased over assessment phases for the high-risk with WM training group but remained relatively stable for the low-risk no WM training group; the center section shows that %WWR did not change appreciably across assessment phases between these groups; the right section shows that UNWR scores improved (increased) over the assessment phases for the high-risk with WM training group, but were approximately constant for the low-risk no WM training group.

The first row of the top section of Table 3 showed that %SS of the high-risk with WM training group differed from the low-risk no WM training group (main effect of group) and that both phase contrasts were affected. The interaction between group and phase was only significant for pre to follow-up (phase contrast 2). There were no significant effects for %WWR for any of the factors (second row of top section of Table 3). Row three of this section of Table 3 showed that, for UNWR, there was a significant main effect of phase contrast 2 (pre to follow-up) and that the interaction of this contrast with group was significant. The interaction arose because the UNWR scores of the low-risk no WM training group did not improve over phases whereas those of the high-risk with WM training group improved. These effects on UNWR may have occurred because the groups differed in level of risk or because this low-risk group did not receive the WM training. This is examined further below when the UNWR scores of the high-risk with WM training and low-risk with WM training groups are compared. There was also an effect of schools Stanford vs. Hatfeild (*p* = 0.045) (Supplementary Table S1).

**TABLE 3:** Summary statistics for sum coding models that predict %SS, %WWR, and UNWR for High-risk with WM training vs. low-risk no WM training groups, High-risk with WM training vs. low-risk with WM training group and Low-risk no WM training vs. low-risk with WM training groups across phases (comparison groups indicated in the far left column).

		Fixed effects	Test group	Phase contrast 1	Phase contrast 2	Phase contrast 1 × Test group	Phase contrast 2 × Test group
High-risk with vs. low-risk no	%SS	Est.	0.372	−0.246	−0.245	−0.193	−0.458
		SE	0.107	0.082	0.084	0.165	0.167
		*t*-value	3.466	−2.986	−2.922	−1.176	−2.734
		*p* (sig.)	0.001 (***)	0.003 (**)	0.003 (**)	0.239 (n.s)	0.006 (**)
	%WWR	Est.	0.401	0.038	−0.019	0.364	0.022
		SE	0.241	0.102	0.103	0.205	0.206
		*t*-value	1.663	0.372	−0.185	1.770	0.106
		*p* (sig.)	0.096 (n.s)	0.710 (n.s)	0.853 (n.s)	0.077 (n.s)	0.915 (n.s)
	UNWR	Est.	−0.622	−0.210	1.811	−0.006	3.566
		SE	1.482	0.660	0.672	1.319	1.343
		*t*-value	−0.420	−0.319	2.696	−0.004	2.655
		*p* (sig.)	0.675 (n.s)	0.750 (n.s)	0.007 (**)	0.997 (n.s)	0.008 (**)
High-risk with vs. low-risk with	%SS	Est.	0.768	−0.287	−0.198	−0.116	−0.539
		SE	0.188	0.106	0.108	0.212	0.215
		*t*-value	4.084	−2.704	−1.844	−0.548	−2.504
		*p* (sig.)	0.000 (***)	0.007 (**)	0.065 (n.s)	0.584 (n.s)	0.012 (*)
	%WWR	Est.	−0.753	0.208	−0.050	−0.477	0.576
		SE	0.819	1.246	0.235	0.492	0.471
		*t-*value	−0.920	0.846	−0.212	−0.969	1.223
		*p* (sig.)	0.358 (n.s)	0.397 (n.s)	0.832 (n.s)	0.333 (n.s)	0.221 (n.s)
	UNWR	Est.	−0.271	−0.567	4.317	−0.200	0.367
		SE	2.251	0.942	0.942	1.884	1.884
		*t-*value	−0.212	−0.602	4.583	−0.106	0.195
		*p* (sig.)	0.904 (n.s)	0.547 (n.s)	0.000 (***)	0.915 (n.s)	0.846 (n.s)
Low-risk with vs. low-risk no	%SS	Est.	−0.214	−0.164	−0.022	−0.030	−0.014
		SE	0.136	0.069	0.070	0.138	0.141
		*t-*value	−1.570	−2.383	−0.321	−0.217	−0.097
		*p* (sig.)	0.116 (n.s)	0.017 (*)	0.748 (n.s)	0.828 (n.s)	0.922 (n.s)
	%WWR	Est.	1.971	0.060	−0.040	0.408	−0.020
		SE	0.432	0.178	0.174	0.357	0.347
		*t-*value	4.565	0.339	−0.230	1.145	−0.058
		*p* (sig.)	0.000 (***)	0.734 (n.s)	0.818 (n.s)	0.252 (n.s)	0.954 (n.s)
	UNWR	Est.	−1.171	−0.342	2.076	−0.249	4.116
		SE	2.524	0.781	0.781	1.562	1.562
		*t-*value	−0.464	−0.438	2.658	−0.160	2.635
		*p* (sig.)	0.643 (n.s)	0.661 (n.s)	0.008 (**)	0.873 (n.s)	0.008 (**)

Analyses for %SS, %WWR, and UNWR scores are indicated in the column second form the left. The factors for which the statistics correspond to, are labeled in the top row. Statistical estimates for the factor and its SE, t-statistic and associated p-values are given for each dependent variable. Table 3 only summarizes the key fixed effect results (see Supplementary Tables S1–S3 for full results). Level of significance: n.s. (p > 0.05), (*p ≤ 0.05), (**p ≤ 0.01), (***p ≤ 0.001).

### Comparison of High-Risk With WM Training Against Low-Risk With WM Training Groups

**FIGURE 2:**
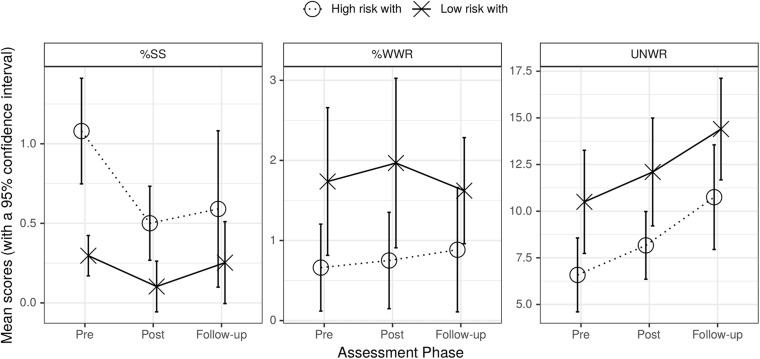
Graph of mean %SS (left), mean %WWR (center), and UNWR scores (right) for high-risk with and low-risk with WM training groups across assessment phases. Bars are standard errors.

Figure 2 gives the results for high-risk and low risk groups who received WM training. The left section shows that %SS decreased over assessment phases for the high-risk with WM training group but remained relatively stable for the low-risk with WM training group (a similar pattern was seen in Figure 1); the middle section shows that %WWR did not change appreciably across assessment phases for either of these groups again as happened in Figure 1; the right section shows that UNWR scores increased over the assessment phases for both groups (unlike what happened when the high-risk with WM training group was compared with the low-risk no WM training group in Figure 1).

The first row of the middle section of Table 3 showed that the statistics for %SS were similar to those in the top section of Table 3 except that phase contrast 2 was not quite significant *(p* = 0.065) and there was an additional effect of age. The second row of this section showed that none of the factors had significant effects on %WWR. For UNWR (third row of the middle section of Table 3) only the main effect of phase contrast 2 was significant. Crucially in all UNWR analyses, the interactions between group and phase contrasts were not significant. Thus, although there were absolute differences between test groups for UNWR, there were no differential changes in these scores over phases between the two groups.

### Comparison of Low-Risk With WM Training Against Low-Risk No WM Training Groups

**FIGURE 3:**
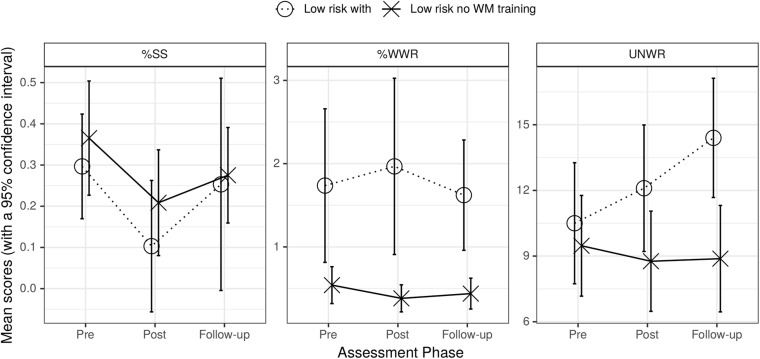
Graph of mean %SS (left), mean %WWR (center), and UNWR scores (right) for low-risk with WM training and low-risk no WM training groups across assessment phases. Bars are standard errors.

The left section of Figure 3 shows that %SS did not differ across the two low-risk groups. The center section shows that the groups differed in absolute value of %WWR but there was little change over phases. The right section shows that the UNWR scores of the low-risk with WM training group increased over phases (improved) after the training whereas the low-risk no WM training showed little change.

The first row of the bottom section of Table 3 showed that only the main effect of pre to post phase (contrast 1) on %SS was significant. Hence, since the interaction was not significant %SS did not change differentially across groups; a similar observation applies with %WWR (here there was only a main effect of test group); UNWR showed a main effect of contrast 2 (pre to follow-up) and an interaction between this contrast and test group. This indicated differential changes in UNWR scores across phases which arose because UNWR scores only improved for the low-risk with WM training group (third row of bottom section of Table 3). Hence, NWR ability appeared to improve in children with low-risk of fluency difficulty when they receive the WM training.

### Individual Group Analyses (Sum Coding and Backward Difference)

Follow-up analyses (sum coding and backward difference) were run next to determine whether the patterns across phases observed in the paired-group analyses were confirmed for individual groups. Since the follow-up analyses were only conducted when the interaction between group and phase was significant in the paired-group analyses, individual group analyses were not conducted for %WWR. Coding 1 involved the pre to post difference for both forms of model to assess replicability, whereas the other coding was specific to the type of model (pre to follow-up for sum coding and post to follow-up for backward difference).

### High-Risk With WM Training Group

The first row of the top section of Table 4 shows that %SS dropped significantly pre to post in both the sum coding and the backward difference analyses. Additionally for %SS, there was a significant drop pre to follow-up (sum coding). UNWR did not change pre to post (both models) but there were significant drops pre to follow-up (sum coding) and post to follow-up (backward difference) as indicated in the top section of Table 4.

**TABLE 4:** Summary statistics for the prediction of %SS and UNWR (sum coding and backward difference coding) for the high-risk with WM training, the low-risk no WM training and low-risk with WM training groups (indicated in the far left column).

			Fixed effects	Phase contrast 1	Phase contrast 2	Phase contrast 3
High risk with training	%SS	Sum coding	Est.	−0.353	−0.453	–
			SE	0.160	0.165	–
			*t*	−2.203	−2.745	–
			*p* (sig.)	0.028 (*)	0.006 (**)	–
		Backward difference coding	Est.	−0.579	–	−0.050
			SE	0.137	–	0.142
			*t*	−4.224	–	−0.356
			*p* (sig.)	0.000 (***)	–	0.722 (n.s)
	UNWR	Sum coding	Est.	−0.667	4.500	–
			SE	1.202	1.202	–
			*t*	−0.555	3.743	–
			*p* (sig.)	0.579 (n.s)	0.000 (***)	–
		Backward difference coding	Est.	1.583	–	2.583
			SE	1.041	–	1.041
			*t*	1.521	–	2.481
			*p* (sig.)	0.128 (n.s)	–	0.013 (*)
Low risk no training	%SS	Sum coding	Est.	−0.112	−0.069	–
			SE	0.074	0.076	–
			*t*	−1.641	−0.916	–
			*p* (sig.)	0.101 (n.s)	0.360 (n.s)	–
		Backward difference coding	Est.	−0.157	–	0.026
			SE	0.064	–	0.065
			*t*	−2.447	–	0.405
			*p* (sig.)	0.014 (*)	–	0.685 (n.s)
	UNWR	Sum coding	Est.	0.073	−0.552	–
			SE	0.740	0.757	–
			*t*	0.099	−0.729	–
			*p* (sig.)	0.921 (n.s)	0.466 (n.s)	–
		Backward difference coding	Est.	−0.203	–	−0.312
			SE	0.646	–	0.646
			*t*	−0.314	–	−0.484
			*p* (sig.)	0.754 (n.s)	–	0.629 (n.s)
Low risk with training	%SS	Sum coding	Est.	−0.229	0.071	–
			SE	0.129	0.129	–
			*t*	−1.770	0.548	–
			*p* (sig.)	0.077 (n.s)	0.584 (n.s)	–
		Backward difference coding	Est.	−0.194	–	0.150
			SE	0.112	–	0.112
			*t*	−1.727	–	1.338
			*p* (sig.)	0.084 (n.s)	–	0.181 (n.s)
	UNWR	Sum coding	Est.	−0.467	4.133	–
			SE	1.463	1.463	–
			t	−0.319	2.825	–
			*p* (sig.)	0.750 (n.s)	0.005 (**)	–
		Backward difference coding	Est.	1.600	–	2.300
			SE	1.267	–	1.267
			*t*	1.263	–	1.815
			*p* (sig.)	0.207 (n.s)	–	0.069 (n.s)

Analyses for %SS and UNWR scores are indicated in the second column from the left, and sum coding and backward difference analyses are indicated in the third column from the left. The factors which the statistics correspond to, are labeled in the top row. Statistical estimates for the factor and its SE, t-statistic and associated p-values are given for each dependent variable. Table 4 only summarizes the fixed effect results (see Supplementary Tables S4–S6 for full results). Level of significance: n.s. (p > 0.05), (*p ≤ 0.05), (**p ≤ 0.01), (***p ≤ 0.001).

### Low-Risk No WM Training Group

There were few changes for the low-risk no WM training group for %SS and for UNWR (middle section of Table 4). The only significant effects for %SS were pre to post for the backward difference model and language group (both models). There were also effects of gender and the two pairs of schools (fixed effects in both models, Supplementary Table S5).

### Low-Risk With WM Training Group

For the low-risk with WM training group, %SS showed no change across any of the phases for both pre to post in both the sum coding and backward difference models (first row of bottom section of Table 4). Therefore, the training did not change the fluency of this group. The only change in UNWR scores was from pre to follow-up (second row of bottom section of Table 4). This was similar to what happened with the high-risk with WM training group (Table 4) except the high-risk group also showed an effect of post to follow-up (backward difference).

## Discussion

### Pairwise-Group Analyses

The data of children working in fixed pairs and assigned to different groups were analyzed pairwise by groups to check whether effects were attributable to the training rather than the passage of time. Sum-coding analyses alone were conducted, which provided statistics for pre to post and pre to follow-up phase effects alone (Table 3).

The critical group by phase interaction is considered first. There was no pre to post (immediate) effect but there was a difference pre to follow-up (long-term) effect when the high-risk with WM training was compared to the low-risk no WM training (Table 3, top section) and with the low-risk with WM training (Table 3, middle section) group. Neither an immediate nor a long-term effect was seen for %SS when the two low-risk groups were compared (Table 3, bottom section). For the fluency measure, the analyses of the group by phase interactions for both low-risk groups suggest that only the high-risk with WM training group responded to the training. The lack of differences on the group by phase interaction across the two low-risk groups for %SS was also consistent with a selective effect on the fluency of the high-risk group. Hence, the overall pattern of these results confirmed the prediction that a change in fluency only occurred for the high-risk group.

The second prediction was that no significant group by phase effects should occur for %WWR as it is an indication of WFD and the training does not address vocabulary issues. This prediction was confirmed since none of the group by phase interactions (for the two contrasts and for the three pairs of groups) were significant (Table 3, all sections).

For UNWR, the two groups who received the training showed no immediate effect across pre and post training, but there were long-term effects (pre to follow-up) relative to the low-risk no WM training group (Table 3, top and bottom sections). This suggests that phonological ability of the high and the low-risk groups benefited from the WM training. Consistent with this conclusion, there was no difference when the two groups who received the training (high-risk/low-risk) were compared (Table 3, middle section). Hence the prediction that the training affects phonological ability of all children (high-risk and low-risk) was supported.

Effects other than the group by phase interactions were examined next. For %SS there was a main effect of test group when the high-risk with WM training and low-risk no WM training groups were compared (Table 3, top section). This main effect was also significant (*p* = 0.0418) when the high-risk and low-risk with WM training were compared (Table 3, middle section), but insignificant when the two low-risk groups were compared (Table 3, bottom section). These differences arose because of the selection criteria (the high-risk group had to have higher %SS than the two low-risk groups). The main effect pre to post was significant for all three group comparisons (Table 3, all sections) and significant or not quite significant for each individual group (Table 4, all sections). Being involved in the study may have benefited all children and could explain why immediate effects of the training were not detected in the group by pre to post phase interaction (discussed above).

The low-risk with WM training group had an unexplained higher rate of %WWR than the low risk no WM training group (main effect of group in Table 3, bottom section). However, as discussed earlier, rate of %WWR did not change at different rates across phases (the group by phase interaction was not significant in any of the %WWR comparisons).

All paired group analyses showed significant increases in UNWR scores between pre and follow-up phases (Table 3). In the individual group analyses, the pre to follow-up effect was significant for high-risk with WM training and low-risk with WM training, but not for low-risk no WM training. Together these results indicated that phonological rehearsal improved for all groups that received the training. There was an additional main effect of group in the analysis of the high-risk and low-risk groups with WM training (Table 3, middle section). The low-risk with WM training group had higher performance but, as the above discussion of the corresponding group by phase interaction shows, rate of change over phases was the same for the two groups.

### Individual Group Analyses

No individual group analyses were conducted for %WWR since there were no significant interactions between groups and phases in any of the pairwise models for this measure. For the remaining measures, an additional contrast was included that allowed post to follow-up differences to be determined. This extra contrast allowed patterns across the three phases to be explored to indicate in detail what the training achieved (the pairwise analyses were restricted to immediate and long-term effects). A significant difference (drop for %SS but rise for UNWR) pre to post and pre to follow-up and no difference post to follow-up indicates a sustained effect (top left quadrant of Table 5); a significant improvement pre to post and a significant reversal post to follow-up, but no difference pre to follow-up indicates an immediate effect alone (bottom left quadrant of Table 5). This pattern shows the training had an effect (pre to post) that was lost (the reversal post to follow-up) and requires no difference pre to follow-up (follow-up returned to its level before the training); a non-significant difference pre to post but significant effects pre to follow-up and post to follow-up indicates a reminiscence effect because it takes some time after the end of training to arise (top left quadrant of Table 5); If all phase comparisons are not significant, the training had no effect (bottom right quadrant of Table 5).

**TABLE 5:** Phase patterns as contingencies pre to post (columns) and pre to follow-up (rows) with additional requirements post to follow-up indicated in the cell entry.

		Pre to post	
		Significant	Not significant
Pre to follow-up	Significant	Sustained (also post to follow-up not significant)	Reminiscence (post to follow-up also has to be significant)
	Not significant	Immediate (post to follow-up also has to show significant increase)	No effect of training (post to follow-up also not significant)

The high-risk group showed a sustained effect across phases for fluency (%SS). Thus, Table 4 (top section) shows significant drops pre-post (contrast 1 in sum coding and backward difference analyses) and pre to follow-up (contrast 2), but no difference post to follow-up (contrast 3). The two low-risk groups (Table 4, middle and bottom sections) showed no significant effect of any of these contrasts except for contrast 1 in the backward difference analysis. The impact of the training on fluency appears to be specific to the high-risk group. This is consistent with the conclusion about %SS in the pairwise analyses. Hence, both pairwise and individual analyses confirmed prediction one.

UNWR scores should increase for both groups who received the training if it improves phonological rehearsal. Both the high-risk and low-risk groups who received the WM training (Table 4, top and bottom sections) showed a reminiscence effect. In both cases the training did not have an effect pre to post training, but did pre to follow-up (retention) and post to follow-up except that post to follow-up was not quite significant (*p* = 0.069) in Table 4 bottom section (low-risk with WM training group). Thus both groups who received the training benefited long term. In contrast, the low-risk group who did not receive the WM training showed no differences across phases (Table 4, middle section).

It was mentioned that sum coding and backward difference analyses were conducted and that each type of analysis gave pre-post estimates. These allowed replicability to be determined. Table 4 shows that all pre post effects (training) where two tests were performed were either both significant or both not significant. The exception was that the low-risk no WM training %SS analyses (Table 4, middle section) had a non-significant effect for sum coding, *p* = 0.101, but was significant for backward difference *p* = 0.014). These probabilities are close and it can therefore be argued that they correspond whichever direction is favored. The high-risk group had a replicable effect on %SS pre to post (Table 4, top section), albeit with a probability close to.05 for sum coding (*p* = 0.028).

### Working memory, Fluency, and Phonological Rehearsal

Comblain (1994) showed training rehearsal strategies improved WM of children with Down syndrome (particularly those aged around five). Rehearsal affected fluency and NWR performance in the present study. Other authors have reported an association between WM and speech fluency (Daneman and Green, 1986; Daneman, 1991). Nevertheless, the specific contribution that WM has on speech fluency remains to be established. The difficulty associated with phonological planning when there is fluency difficulty (Howell, 2010b) appears to be moderated as verbal WM skills improve. This could make speech plans for difficult words (usually content) available for speech output earlier by facilitating retrieval which, in turn, prevents fluency breakdown. The findings with UNWR (all children whatever their fluency risk showed improved scores when given the training) support the view that word rehearsal in the WM task improves young children’s articulatory ability.

The results also showed that there were no significant changes in %WWR over the assessment phases for both groups. This suggests that WWR was not affected by the WM training and once again that the training works on an articulatory, rather than lexical, level. Hence, fluency difficulty and WFD appear to have different etiologies that require different types of training. The present procedure for fluency difficulty could be offered in schools. Whilst it was emphasized that the effects of any training had to be at least neutral if delivered to fluent children, it was actually found to benefit phonological ability of children at low risk of fluency difficulty, commending its use in schools. A WFD training procedure is being developed (Howell et al., 2017b). If WWR results from difficulties in lexical retrieval (Bada, 2010), a WFD training procedure needs to improve vocabulary and, given the high proportion of children with EAL in this sample and in UK schools in general, to ensure that any training is appropriate for children with diverse language backgrounds.

### Limitations

The overall pattern of results in both the pairwise and individual groups analyses support the hypotheses that the WM training improves the fluency of children at risk of fluency difficulty, that the training improves the phonological rehearsal skills of all children but that WFD is not affected. Whilst the majority of the statistics were consistent with this description, effects that should have been significant were sometimes not quite significant and effects that should not have been significant only approached significance. One example is that the individual group analysis for low-risk with training on UNWR for phase contrast 3 had *p* = 0.069 (Table 4, bottom section). A second is that although it was stated that the replication analyses for contrast 1 all had the same significant/non-significant pattern across sum coding and backward difference *ps* were 0.101 in the sum coding and 0.014 in the backward difference analyses for %SS of the low-risk no WM training group (Table 4, middle section). Pre versus post can give different results depending what other contrasts it is paired with (sum-coded and backward difference models). A contrast included in different forms of model can result in variance being partitioned in different ways, which can make the same contrast significant in one form of analysis but insignificant in the other. This may be operating in the UNWR analysis for contrast 1 in Table 4, top section (high-risk with WM training group where the sum coding analysis has a *p* of 0.579 whereas the backward difference analysis has a *p* of 0.128. In these cases, caution needs to be exercised. It could be argued that the children in the control condition who received a non-WM version of the memory games might conceivably have been less engaged as their version of the task was not as challenging. No measure of engagement was taken, but the experimenters reported that children in the condition that did not involve WM training appeared to be as involved in the tasks as children on the other groups.

Further work is needed with larger-sized samples to estimate reliable effect sizes and to assess clinical importance of the WM training procedure. Our findings do, nonetheless, allow a preliminary estimate of the effect sizes for the significant improvement across phases for fluency (%SS) and phonological rehearsal (UNWR) (Table 4). First, the sustained effect of training on fluency was found with the high risk group. %SS decreased in the range of 0.353–0.579% from pre to post. A threshold of 3% is frequently used as a threshold for distinguishing fluent speakers from speakers who stutter (Yairi and Ambrose, 2005). Riley’s (2009) Figure 2.2 shows that a 1% change in %SS would move a child from above, to below, the 3% threshold (change from “stutters” to “fluent”). Based on this, a reduction of around 0.5% in %SS appears clinically relevant. Second, the reminiscence effect of training on UNWR was found with both the high risk, and the low-risk, groups. UNWR increased from pre- to follow-up by 4.133 points (low risk) and 4.500 points (high risk), and from post- to follow-up by 2.300 points (low risk) and 2.583 points (high risk). Hakim and Ratner’s (2004) Table 2 shows that the difference between mean number of stimuli correct across children who stutter and those who are fluent is 0.5 for two-syllable non-words. Hence, the improvement in scores of around 2.3 stimuli (the most conservative estimate) in the current study is higher than this difference value of 0.5 and could also indicate potentially clinically-relevant effects on fluency.

The ability to correctly recall the stimuli in reverse order improved gradually for the majority of the children. However, some children continued to struggle even with the easy two-item material. When such children were presented with three-item material subsequently, they often made errors and this could weaken the effects of the training. A possible solution is provided by Holmes et al. (2009), who demonstrated that adaptive WM training led to greater improvements on various WM measures as compared to non-adaptive training. An adaptive training procedure could lead to benefits of the training for most children, as the training would adjust to the individual abilities of each child. Some attention was given to ensuring the training was reliably and consistently delivered. Future work should include video recording of sessions and statistical measure for areas 2–4 under training fidelity.

## Conclusion

This study addressed a WM study for, children with fluency difficulty. Children were screened for risk of fluency difficulty using Howell et al.’s (2017a) procedure. The study was based on a WM training task. This led to marked improvements in speech fluency of children at high-risk and improved phonological ability of children whatever their risk. The study provides a suitable preliminary procedure that schools could perform. Schools may report children to SLPs immediately or after preliminary training if they do not respond to the training. Together in-school and SLP training could provide continuing and phased support for all children identified as at risk of fluency difficulty. The training allows schools to address a range of fluency difficulties at an early stage, which would otherwise affect the children’s academic performance.

## Data Availability Statement

The raw data supporting the conclusions of this article will be made available by the authors, without undue reservation, to any qualified researcher.

## Ethics Statement

The studies involving human participants were reviewed and approved by the University College London (0078/004). Written informed consent from the participants’ legal guardian/next of kin was not required to participate in this study in accordance with the national legislation and the institutional requirements.

## Author Contributions

PH, JH, and KT designed the research. PH, LC, KY, HT, and KT performed the research. PH, LC, KY, TW, and KT analyzed the data. PH and KT wrote the manuscript. All authors contributed to the article and approved the submitted version.

## Conflict of Interest

The authors declare that the research was conducted in the absence of any commercial or financial relationships that could be construed as a potential conflict of interest. The reviewer KM declared a shared affiliation, with no collaboration, with several of the authors, PH, LC, KY, HT, TW, and JH, to the handling editor at the time of the review.

## Footnotes

1 See https://www.ipswich.gov.uk/sites/default/files/State%20of%20Ipswich%20 AMReport%20(v1%201)%202014.pdf

2 See https://www.hackney.gov.uk/media/2664/Facts-and-figures/pdf/facts-and-figures

3 See http://www.merton.gov.uk/jsna_summary_document_2015_final.pdf

4 The 20 languages UNWR was originally developed for are English, Polish, Romanian, European Portuguese, Bulgarian, Serbo-Croat-Bosnian, Czech, Dutch, French, German, Hungarian, Slovene, Swedish, Danish, Norwegian, Russian, Latvian, Ukrainian, Urdu-Hindi and Bengali. The UNWR tests these 20 languages equitably. UNWR as well as CNRep stimuli (Gathercole et al., 1994) are available at www.fistproject.org. Three children who spoke Turkish and Pashto were also included based on sample size considerations and because the design constraints on UNWR apply to these languages too.

5 One child had %SS of zero because he did not produce any speech sample. Nevertheless he was included in the high-risk WM training group because the child and the school requested inclusion in the study.

6 The individual group models for the low-risk with WM training group has only one school. Hence “school” was not a random factor.
