# Supplementary material for: Does Working-Memory Training Given to Reception-Class Children Improve the Speech of Children at Risk of Fluency Difficulty?
Source: Front Psychol. 2020 Nov 17;11:568867. doi: 10.3389/fpsyg.2020.568867 (PMC7718024; doi:10.3389/fpsyg.2020.568867)
Supplement: Supplementary file 1 [file Table_1.DOCX]

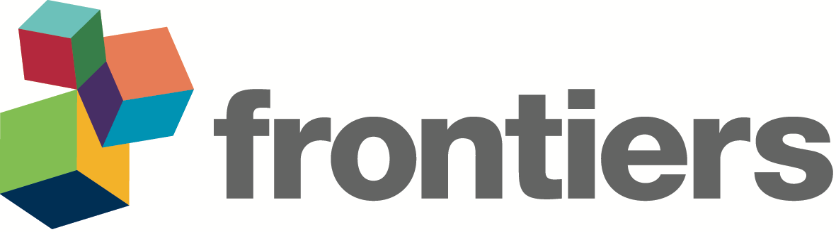


***Supplementary Material***

**1 SUPPLEMENTARY TABLES**

**1.1**

|  | ***%SS*** | | | | ***%WWR*** | | | | ***UNWR*** | | | |
| --- | --- | --- | --- | --- | --- | --- | --- | --- | --- | --- | --- | --- |
| **Fixed Effects** | ***Est.*** | ***SE*** | ***t-value*** | ***p (sig.)*** | ***Est.*** | ***SE*** | ***t-value*** | ***p (sig.)*** | ***Est.*** | ***SE*** | ***t-value*** | ***p (sig.)*** |
| *(Intercept)* | 0.463 | 0.055 | 8.425 | 0.000  (***) | 0.539 | 0.124 | 4.342 | 0.000  (***) | 8.039 | 0.764 | 10.523 | 0.000  (***) |
| Test group  (Low-risk no training vs. High-risk) | 0.372 | 0.107 | 3.466 | 0.001  (***) | 0.401 | 0.241 | 1.663 | 0.096  (n.s) | -0.622 | 1.482 | -0.420 | 0.675  (n.s) |
| Phase contrast 1  (Pre to Post) | -0.246 | 0.082 | -2.986 | 0.003  (**) | 0.038 | 0.102 | 0.372 | 0.710  (n.s) | -0.210 | 0.660 | -0.319 | 0.750  (n.s) |
| Phase contrast 2  (Pre to follow-up) | -0.245 | 0.084 | -2.922 | 0.003  (**) | -0.019 | 0.103 | -0.185 | 0.853  (n.s) | 1.811 | 0.672 | 2.696 | 0.007  (**) |
| Phase contrast 1 x Test group | -0.193 | 0.165 | -1.176 | 0.239  (n.s) | 0.364 | 0.205 | 1.770 | 0.077  (n.s) | -0.006 | 1.319 | -0.004 | 0.997  (n.s) |
| Phase contrast 2 x Test group | -0.458 | 0.167 | -2.734 | 0.006  (**) | 0.022 | 0.206 | 0.106 | 0.915  (n.s) | 3.566 | 1.343 | 2.655 | 0.008  (**) |
| Language group  (EngMono. Vs. EAL) | 0.089 | 0.145 | 0.611 | 0.541  (n.s) | 0.077 | 0.334 | 0.231 | 0.817  (n.s) | -1.328 | 2.050 | -0.648 | 0.517  (n.s) |
| Gender (Female vs. Male) | 0.013 | 0.143 | 0.092 | 0.926  (n.s) | 0.237 | 0.326 | 0.727 | 0.467  (n.s) | -1.932 | 2.004 | -0.964 | 0.335  (n.s) |
| Age | 0.097 | 0.064 | 1.508 | 0.131  (n.s) | 0.199 | 0.150 | 1.324 | 0.185  (n.s) | 1.707 | 0.919 | 1.857 | 0.063  (n.s) |
| School  (Stanford vs. Hatfeild) | -0.086 | 0.196 | -0.440 | 0.660  (n.s) | 0.371 | 0.443 | 0.839 | 0.402  (n.s) | 1.584 | 2.727 | 0.581 | 0.561  (n.s) |
| School  (Stanford vs. Priory) | 0.093 | 0.237 | 0.392 | 0.695  (n.s) | 0.401 | 0.540 | 0.743 | 0.457  (n.s) | 6.673 | 3.326 | 2.006 | 0.045  (*) |
| School  (Stanford vs. St. Helen's) | 0.202 | 0.170 | 1.187 | 0.235  (n.s) | 0.348 | 0.380 | 0.914 | 0.360  (n.s) | -3.967 | 2.332 | -1.701 | 0.089  (n.s) |
| **Random Effect** | ***Variance*** | | ***SD*** | | ***Variance*** | | ***SD*** | | ***Variance*** | | ***SD*** | |
| Participant | 0.037 | | 0.193 | | 0.306 | | 0.553 | | 11.367 | | 3.371 | |
| Pairing | 0.004 | | 0.066 | | 0.000 | | 0.000 | | 0.000 | | 0.000 | |
| Residual | 0.071 | | 0.266 | | 0.105 | | 0.323 | | 4.502 | | 2.122 | |
| ***# data points dropped*** | 1 | | | | 2 | | | | 2 | | | |
| Level of significance: n.s. (p > 0.05), * (p ≤ 0.05), ** (p ≤ 0.01), *** (p ≤ 0.001) | | | | | | | | | | | | |

**Table S1. High-risk with WM training, versus low-risk no WM training groups.** Summary statistics for sum coding models that predict %SS, %WWR and UNWR for low-risk no WM training versus high-risk with WM training groups across phases. %SS, %WWR and UNWR scores can be identified from the top row of the table. The factors which the statistics correspond to, are labeled in the left-most column. Statistical estimates for the factor and its SE, t statistic and associated p values are given for each dependent variable. The first half of the table and the second half of the table give the statistics of the fixed effects and random effects correspondingly. The last row of the table gives the statistical values associated with the symbols used in the main table. The number of data points dropped refers to the number of outliers with residuals more than 2.5 SD from the mean were removed after the initial model was built.

**1.2**

|  | **%SS** | | | | **%WWR** | | | | **UNWR** | | | |
| --- | --- | --- | --- | --- | --- | --- | --- | --- | --- | --- | --- | --- |
| **Fixed Effects** | ***Est.*** | ***SE*** | ***t-value*** | ***p (sig.)*** | ***Est.*** | ***SE*** | ***t-value*** | ***p (sig.)*** | ***Est.*** | ***SE*** | ***t-value*** | ***p (sig.)*** |
| *(Intercept)* | 0.440 | 0.065 | 6.800 | 0.000  (***) | 1.242 | 0.250 | 4.968 | 0.000  (***) | 9.814 | 0.871 | 11.262 | 0.000  (***) |
| Test group  (Low-risk with training vs. High-risk) | 0.768 | 0.188 | 4.084 | 0.000  (***) | -0.753 | 0.819 | -0.920 | 0.358  (n.s) | -0.271 | 2.251 | -0.212 | 0.904  (n.s) |
| Phase contrast 1  (Pre to Post) | -0.287 | 0.106 | -2.704 | 0.007  (**) | 0.208 | 1.246 | 0.846 | 0.397  (n.s) | -0.567 | 0.942 | -0.602 | 0.547  (n.s) |
| Phase contrast 2  (Pre to follow-up) | -0.198 | 0.108 | -1.844 | 0.065  (n.s) | -0.050 | 0.235 | -0.212 | 0.832  (n.s) | 4.317 | 0.942 | 4.583 | 0.000  (***) |
| Phase contrast 1 x Test group | -0.116 | 0.212 | -0.548 | 0.584  (n.s) | -0.477 | 0.492 | -0.969 | 0.333  (n.s) | -0.200 | 1.884 | -0.106 | 0.915  (n.s) |
| Phase contrast 2 x Test group | -0.539 | 0.215 | -2.504 | 0.012  (*) | 0.576 | 0.471 | 1.223 | 0.221  (n.s) | 0.367 | 1.884 | 0.195 | 0.846  (n.s) |
| Language group  (EngMono. Vs. EAL) | 0.125 | 0.127 | 0.985 | 0.324  (n.s) | 0.005 | 0.533 | 0.009 | 0.993  (n.s) | -2.880 | 1.575 | -1.829 | 0.067  (n.s) |
| Gender (Female vs. Male) | 0.143 | 0.135 | 1.066 | 0.286  (n.s) | 0.674 | 0.552 | 1.221 | 0.222  (n.s) | -1.125 | 1.671 | -0.673 | 0.501  (n.s) |
| Age | 0.214 | 0.087 | 2.449 | 0.014  (*) | 0.197 | 0.414 | 0.477 | 0.633  (n.s) | 1.871 | 0.949 | 1.971 | 0.049  (*) |
| School  (Stanford vs. Hatfeild) | -0.232 | 0.402 | -0.577 | 0.564  (n.s) | -0.174 | 1.626 | -0.107 | 0.915  (n.s) | 2.615 | 5.311 | 0.492 | 0.622  (n.s) |
| School  (Stanford vs. Priory) | 0.020 | 0.307 | 0.066 | 0.947  (n.s) | -0.028 | 1.185 | -0.024 | 0.981  (n.s) | 2.656 | 4.039 | 0.658 | 0.511  (n.s) |
| School  (Stanford vs. St. Helen's) | 0.447 | 0.259 | 1.726 | 0.084  (n.s) | 0.759 | 0.983 | 0.772 | 0.440  (n.s) | -2.075 | 3.478 | -0.597 | 0.551  (n.s) |
| **Random Effect** | ***Variance*** | | ***SD*** | | ***Variance*** | | ***SD*** | | ***Variance*** | | ***SD*** | |
| Participant | 0.000 | | 0.000 | | 0.720 | | 0.848 | | 1.228 | | 1.108 | |
| Pairing | 0.024 | | 0.154 | | 0.000 | | 0.0000 | | 6.072 | | 2.464 | |
| Residual | 0.092 | | 0.303 | | 0.440 | | 0.663 | | 7.258 | | 2.694 | |
| ***# data points dropped*** | 1 | | | | 2 | | | | 0 | | | |
| Level of significance: n.s. (p > 0.05), * (p ≤ 0.05), ** (p ≤ 0.01), *** (p ≤ 0.001) | | | | | | | | | | | | |

**Table S2. High-risk versus low-risk with WM training groups**. Summary statistics for sum coding models that predict %SS, %WWR and UNWR for low-risk with WM training versus high-risk with WM training groups across phases. %SS, %WWR and UNWR scores can be identified from the top row of the table. The factors which the statistics correspond to, are labeled in the left-most column. Statistical estimates for the factor and its SE, t statistic and associated p values are given for each dependent variable. The first half of the table and the second half of the table give the statistics of the fixed effects and random effects correspondingly The last row of the table gives the statistical values associated with the symbols used in the main table. The number of data points dropped refers to the number of outliers with residuals more than 2.5 SD from the mean were removed after the initial model was built.

**1.3**

|  | ***%SS*** | | | | ***%WWR*** | | | | ***UNWR*** | | | | |
| --- | --- | --- | --- | --- | --- | --- | --- | --- | --- | --- | --- | --- | --- |
| **Fixed Effects** | ***Est.*** | ***SE*** | ***t-value*** | ***p (sig.)*** | ***Est.*** | ***SE*** | ***t-value*** | ***p (sig.)*** | ***Est.*** | ***SE*** | ***t-value*** | ***p (sig.)*** |  |
| *(Intercept)* | 0.259 | 0.047 | 5.515 | 0.000  (***) | 1.251 | 0.161 | 7.750 | 0.000  (***) | 8.970 | 0.869 | 10.321 | 0.000  (***) |  |
| Test group  (Low-risk no training vs. Low-risk training) | -0.214 | 0.136 | -1.570 | 0.116  (n.s) | 1.971 | 0.432 | 4.565 | 0.000  (***) | -1.171 | 2.524 | -0.464 | 0.643  (n.s) |  |
| Phase contrast 1  (Pre to Post) | -0.164 | 0.069 | -2.383 | 0.017  (*) | 0.060 | 0.178 | 0.339 | 0.734  (n.s) | -0.342 | 0.781 | -0.438 | 0.661  (n.s) |  |
| Phase contrast 2  (Pre to follow-up) | -0.022 | 0.070 | -0.321 | 0.748  (n.s) | -0.040 | 0.174 | -0.230 | 0.818  (n.s) | 2.076 | 0.781 | 2.658 | 0.008  (**) |  |
| Phase contrast 1 x Test group | -0.030 | 0.138 | -0.217 | 0.828  (n.s) | 0.408 | 0.357 | 1.145 | 0.252  (n.s) | -0.249 | 1.562 | -0.160 | 0.873  (n.s) |  |
| Phase contrast 2 x Test group | -0.014 | 0.141 | -0.097 | 0.922  (n.s) | -0.020 | 0.347 | -0.058 | 0.954  (n.s) | 4.116 | 1.562 | 2.635 | 0.008  (**) |  |
| Language group  (EngMono. Vs. EAL) | -0.051 | 0.097 | -0.525 | 0.600  (n.s) | 0.303 | 0.308 | 0.984 | 0.325  (n.s) | -0.850 | 1.810 | -0.469 | 0.639  (n.s) |  |
| Gender (Female vs. Male) | -0.063 | 0.090 | -0.700 | 0.484  (n.s) | 0.332 | 0.292 | 1.137 | 0.256  (n.s) | 0.505 | 1.662 | 0.304 | 0.761  (n.s) |  |
| Age | 0.029 | 0.076 | 0.384 | 0.701  (n.s) | -0.329 | 0.216 | -1.526 | 0.127  (n.s) | 3.616 | 1.409 | 2.566 | 0.010  (**) |  |
| School  (Stanford vs. Hatfeild) | -0.165 | 0.167 | -0.990 | 0.322  (n.s) | 0.243 | 0.575 | 0.423 | 0.673  (n.s) | 3.073 | 3.110 | 0.988 | 0.323  (n.s) |  |
| School  (Stanford vs. Priory) | -0.243 | 0.222 | -1.096 | 0.273  (n.s) | -0.529 | 0.744 | -0.710 | 0.477  (n.s) | 9.403 | 4.128 | 2.278 | 0.023  (*) |  |
| School  (Stanford vs. St. Helen's) | -0.104 | 0.178 | -0.587 | 0.557  (n.s) | 0.418 | 0.638 | 0.655 | 0.512  (n.s) | -2.493 | 3.293 | -0.757 | 0.449  (n.s) |  |
| **Random Effect** | ***Variance*** | | ***SD*** | | ***Variance*** | | ***SD*** | | ***Variance*** | | ***SD*** | | |
| Participant | 0.018 | | 0.133 | | 0.125 | | 0.354 | | 9.255 | | 3.042 | | |
| Pairing | 0.000 | | 0.000 | | 0.147 | | 0.383 | | 0.000 | | 0.000 | | |
| Residual | 0.044 | | 0.210 | | 0.260 | | 0.510 | | 5.727 | | 2.393 | | |
| ***# data points dropped*** | 1 | | | | 3 | | | | 1 | | | | |
| Level of significance: n.s. (p > 0.05), * (p ≤ 0.05), ** (p ≤ 0.01), *** (p ≤ 0.001) | | | | | | | | | | | | | |

**Table S3. Low-risk with WM training versus low-risk no WM training groups.** Summary statistics for sum coding models that predict %SS, %WWR and UNWR for low-risk no WM training versus low-risk with WM training groups across phases. %SS, %WWR and UNWR scores can be identified from the top row of the table. The factors to which the statistics correspond are labeled in the left-most column. Statistical estimates for the factor and its SE, t statistic and associated p values are given for each dependent variable. The first half of the table and the second half of the table give the statistics of the fixed effects and random effects correspondingly. The last row of the table gives the statistical values associated with the symbols used in the main table. The number of data points dropped refers to the number of outliers with residuals more than 2.5 SD from the mean were removed after the initial model was built.

**1.4**

|  | ***%SS*** | | | | | | | | ***UNWR*** | | | | | | | |
| --- | --- | --- | --- | --- | --- | --- | --- | --- | --- | --- | --- | --- | --- | --- | --- | --- |
|  | ***Sum coding*** | | | | ***Backward difference coding*** | | | | ***Sum coding*** | | | | ***Backward difference coding*** | | | |
| **Fixed Effects** | ***Est.*** | ***SE*** | ***t*** | ***p (sig.)*** | ***Est.*** | ***SE*** | ***t*** | ***p (sig.)*** | ***Est.*** | ***SE*** | ***t*** | ***p (sig.)*** | ***Est.*** | ***SE*** | ***t*** | ***p (sig.)*** |
| (Intercept) | 0.661 | 0.179 | 3.684 | 0.000  (***) | 0.661 | 0.179 | 3.684 | 0.000  (***) | 7.319 | 1.499 | 4.881 | 0.000  (***) | 7.319 | 1.499 | 4.881 | 0.000  (***) |
| Phase contrast 1  (Pre to Post) | -0.353 | 0.160 | -2.203 | 0.028  (*) | -0.579 | 0.137 | -4.224 | 0.000  (***) | -0.667 | 1.202 | -0.555 | 0.579  (n.s) | 1.583 | 1.041 | 1.521 | 0.128  (n.s) |
| Phase contrast 2  (Pre to follow-up) | -0.453 | 0.165 | -2.745 | 0.006  (**) |  |  |  |  | 4.500 | 1.202 | 3.743 | 0.000  (***) |  |  |  |  |
| Phase contrast 3  (Post to follow-up) |  |  |  |  | -0.050 | 0.142 | -0.356 | 0.722  (n.s) |  |  |  |  | 2.583 | 1.041 | 2.481 | 0.013  (*) |
| Language group  (EngMono. Vs. EAL) | 0.247 | 0.398 | 0.620 | 0.535  (n.s) | 0.247 | 0.398 | 0.620 | 0.535  (n.s) | -2.598 | 2.221 | -1.170 | 0.242  (n.s) | -2.598 | 2.221 | -1.170 | 0.242  (n.s) |
| Gender (Female vs. Male) | 0.276 | 0.517 | 0.535 | 0.593  (n.s) | 0.276 | 0.517 | 0.535 | 0.593  (n.s) | -6.284 | 4.409 | -1.425 | 0.154  (n.s) | -6.284 | 4.409 | -1.425 | 0.154  (n.s) |
| Age | 0.214 | 0.138 | 1.546 | 0.122  (n.s) | 0.214 | 0.138 | 1.546 | 0.112  (n.s) | 0.569 | 0.752 | 0.757 | 0.449  (n.s) | 0.569 | 0.752 | 0.757 | 0.449  (n.s) |
| School  (Stanford vs. Hatfeild) | 0.026 | 0.741 | 0.035 | 0.972  (n.s) | 0.026 | 0.740 | 0.035 | 0.972  (n.s) | -1.630 | 6.315 | -0.258 | 0.796  (n.s) | -1.630 | 6.315 | -0.258 | 0.796  (n.s) |
| School  (Stanford vs. Priory) | 0.017 | 0.622 | 0.027 | 0.978  (n.s) | 0.017 | 0.622 | 0.027 | 0.978  (n.s) | 8.681 | 6.404 | 1.255 | 0.175  (n.s) | 8.681 | 6.404 | 1.255 | 0.175  (n.s) |
| School  (Stanford vs. St. Helen's) | 0.370 | 0.404 | 0.916 | 0.360  (n.s) | 0.370 | 0.404 | 0.916 | 0.360  (n.s) | -1.301 | 3.665 | -0.335 | 0.723  (n.s) | -1.301 | 3.665 | -0.335 | 0.723  (n.s) |
| **Random Effect** | ***Variance*** | | | | ***SD*** | | | | ***Variance*** | | | | ***SD*** | | | |
| Participant | 0.059 | | | | 0.242 | | | | 0.000 | | | | 0.000 | | | |
| Pairing | 0.000 | | | | 0.004 | | | | 7.890 | | | | 2.809 | | | |
| Residual | 0.113 | | | | 0.336 | | | | 6.503 | | | | 2.550 | | | |
| **# data points dropped** | 1 | | | | | | | | 0 | | | | | | | |
| Level of significance: n.s. (p > 0.05), * (p ≤ 0.05), ** (p ≤ 0.01), *** (p ≤ 0.001) | | | | | | | | | | | | | | | | |

**Table S4.** Summary statistics for the prediction of %SS and UNWR (sum coding and backward difference coding) for the high-risk with WM training group. %SS and UNWR scores can be identified from the top row of the table and sum coding versus backward difference from the next row. The factors which the statistics correspond to, are labeled in the left-most column. Statistical estimates for the factor and its SE, t statistic and associated p values are given for each dependent variable. The first half of the table and the second half of the table give the statistics of the fixed effects and random effects correspondingly. The last row of the table gives the statistical values associated with the symbols used in the main table. The number of data points dropped refers to the number of outliers with residuals more than 2.5 SD from the mean were removed after the initial model was built.

**1.5**

|  | ***%SS*** | | | | | | | | ***UNWR*** | | | | | | | |
| --- | --- | --- | --- | --- | --- | --- | --- | --- | --- | --- | --- | --- | --- | --- | --- | --- |
|  | ***Sum coding*** | | | | ***Backward difference coding*** | | | | ***Sum coding*** | | | | ***Backward difference coding*** | | | |
| **Fixed Effects** | ***Est.*** | ***SE*** | ***t*** | ***p (sig.)*** | ***Est.*** | ***SE*** | ***t*** | ***p (sig.)*** | ***Est.*** | ***SE*** | ***t*** | ***p (sig.)*** | ***Est.*** | ***SE*** | ***t*** | ***p (sig.)*** |
| (Intercept) | 0.385 | 0.041 | 9.461 | 0.000  (***) | 0.385 | 0.041 | 9.461 | 0.000  (***) | 7.363 | 1.330 | 5.534 | 0.000  (***) | 7.363 | 1.330 | 5.534 | 0.000  (***) |
| Phase contrast 1  (Pre to Post) | -0.112 | 0.074 | -1.641 | 0.101  (n.s) | -0.157 | 0.064 | -2.447 | 0.014  (*) | 0.073 | 0.740 | 0.099 | 0.921  (n.s) | -0.203 | 0.646 | -0.314 | 0.754  (n.s) |
| Phase contrast 2  (Pre to follow-up) | -0.069 | 0.076 | -0.916 | 0.360  (n.s) |  |  |  |  | -0.552 | 0.757 | -0.729 | 0.466  (n.s) |  |  |  |  |
| Phase contrast 3  (Post to follow-up) |  |  |  |  | 0.026 | 0.065 | 0.405 | 0.685  (n.s) |  |  |  |  | -0.312 | 0.646 | -0.484 | 0.629  (n.s) |
| Language group  (EngMono. Vs. EAL) | -0.336 | 0.108 | -3.119 | 0.002  (**) | -0.336 | 0.108 | -3.119 | 0.002  (**) | 1.139 | 3.524 | 0.323 | 0.746  (n.s) | 1.139 | 3.524 | 0.323 | 0.746  (n.s) |
| Gender (Female vs. Male) | -0.438 | 0.104 | -4.213 | 0.000  (***) | -0.438 | 0.104 | -4.213 | 0.000  (***) | 1.229 | 3.387 | 0.363 | 0.717  (n.s) | 1.229 | 3.387 | 0.363 | 0.717  (n.s) |
| Age | 0.068 | 0.039 | 1.726 | 0.084  (n.s) | 0.068 | 0.039 | 1.726 | 0.084  (n.s) | 2.402 | 1.286 | 1.868 | 0.062  (n.s) | 2.402 | 1.286 | 1.868 | 0.062  (n.s) |
| School  (Stanford vs. Hatfeild) | -0.235 | 0.114 | -2.063 | 0.039  (*) | -0.235 | 0.114 | -2.063 | 0.039  (*) | 3.431 | 3.733 | 0.919 | 0.358  (n.s) | 3.431 | 3.733 | 0.919 | 0.358  (n.s) |
| School  (Stanford vs. Priory) | -0.340 | 0.164 | -2.078 | 0.037  (*) | -0.340 | 0.164 | -2.078 | 0.037  (*) | 11.568 | 5.349 | 2.163 | 0.031  (*) | 11.568 | 5.349 | 2.163 | 0.031  (*) |
| School  (Stanford vs. St. Helen's) | -0.384 | 0.136 | -2.820 | 0.005  (**) | -0.384 | 0.136 | -2.820 | 0.005  (**) | -2.839 | 4.457 | -0.637 | 0.524  (n.s) | -2.839 | 4.457 | -0.637 | 0.524  (n.s) |
| **Random Effect** | ***Variance*** | | | | ***SD*** | | | | ***Variance*** | | | | ***SD*** | | | |
| Participant | 0.003 | | | | 0.053 | | | | 14.280 | | | | 3.778 | | | |
| Pairing | 0.000 | | | | 0.000 | | | | 0.000 | | | | 0.000 | | | |
| Residual | 0.035 | | | | 0.187 | | | | 3.391 | | | | 1.842 | | | |
| **# data points dropped** | 1 | | | | | | | | 2 | | | | | | | |
| Level of significance: n.s. (p > 0.05), * (p ≤ 0.05), ** (p ≤ 0.01), *** (p ≤ 0.001) | | | | | | | | | | | | | | | | |

**Table S5.** Summary statistics for the prediction of %SS and UNWR (sum coding and backward difference coding) for the low-risk no WM training group. %SS and UNWR scores can be identified from the top row of the table and sum coding versus backward difference from the next row. The factors to which the statistics correspond, are labeled in the left-most column. Statistical estimates for the factor and its SE, t statistic and associated p values are given for each dependent variable. The last row of the table gives the statistical values associated with the symbols used in the main table. The number of data points dropped refers to the number of outliers with residuals more than 2.5 SD from the mean were removed after the initial model was built.

**1.6**

|  | ***%SS*** | | | | | | | | ***UNWR*** | | | | | | | |
| --- | --- | --- | --- | --- | --- | --- | --- | --- | --- | --- | --- | --- | --- | --- | --- | --- |
|  | ***Sum coding*** | | | | ***Backward difference coding*** | | | | ***Sum coding*** | | | | ***Backward difference coding*** | | | |
| **Fixed Effects** | ***Est.*** | ***SE*** | ***t*** | ***p (sig.)*** | ***Est.*** | ***SE*** | ***t*** | ***p (sig.)*** | ***Est.*** | ***SE*** | ***t*** | ***p (sig.)*** | ***Est.*** | ***SE*** | ***t*** | ***p (sig.)*** |
| (Intercept) | 0.140 | 0.148 | 0.949 | 0.343  (n.s) | 0.140 | 0.148 | 0.949 | 0.343  (n.s) | 7.504 | 1.999 | 3.754 | 0.000  (***) | 7.504 | 1.999 | 3.754 | 0.000  (***) |
| Phase contrast 1  (Pre to Post) | -0.229 | 0.129 | -1.770 | 0.077  (n.s) | -0.194 | 0.112 | -1.727 | 0.084  (n.s) | -0.467 | 1.463 | -0.319 | 0.750  (n.s) | 1.600 | 1.267 | 1.263 | 0.207  (n.s) |
| Phase contrast 2  (Pre to follow-up) | 0.071 | 0.129 | 0.548 | 0.584  (n.s) |  |  |  |  | 4.133 | 1.463 | 2.825 | 0.005  (**) |  |  |  |  |
| Phase contrast 3  (Post to follow-up) |  |  |  |  | 0.150 | 0.112 | 1.338 | 0.181  (n.s) |  |  |  |  | 2.300 | 1.267 | 1.815 | 0.069  (n.s) |
| Language group  (EngMono. Vs. EAL) | 0.044 | 0.136 | 0.322 | 0.747  (n.s) | 0.044 | 0.136 | 0.322 | 0.747  (n.s) | -2.604 | 2.002 | -1.301 | 0.193  (n.s) | -2.604 | 2.002 | -1.301 | 0.193  (n.s) |
| Gender (Female vs. Male) | 0.164 | 0.112 | 1.464 | 0.143  (n.s) | 0.164 | 0.112 | 1.464 | 0.143  (n.s) | -0.380 | 1.582 | -0.240 | 0.810  (n.s) | -0.380 | 1.582 | -0.240 | 0.810  (n.s) |
| Age | 0.084 | 0.138 | 0.611 | 0.541  (n.s) | 0.084 | 0.138 | 0.611 | 0.541  (n.s) | 4.006 | 1.789 | 2.239 | 0.025  (*) | 4.006 | 1.789 | 2.239 | 0.025  (*) |
| **Random Effect** | ***Variance*** | | | | ***SD*** | | | | ***Variance*** | | | | ***SD*** | | | |
| Participant | 0.000 | | | | 0.000 | | | | 0.397 | | | | 0.630 | | | |
| Pairing | 0.009 | | | | 0.097 | | | | 3.926 | | | | 1.981 | | | |
| Residual | 0.063 | | | | 0.251 | | | | 8.026 | | | | 2.833 | | | |
| **# data points dropped** | 0 | | | | | | | | 0 | | | | | | | |
| Level of significance: n.s. (p > 0.05), * (p ≤ 0.05), ** (p ≤ 0.01), *** (p ≤ 0.001) | | | | | | | | | | | | | | | | |

**Table S6**. Summary statistics for the prediction of %SS and UNWR (sum coding and backward difference coding) for the low-risk with WM training group. %SS and UNWR scores can be identified from the top row of the table and sum coding versus backward difference from the next row. The factors to which the statistics correspond, are labeled in the left-most column. Statistical estimates for the factor and its SE, t statistic and associated p values are given for each dependent variable. The last row of the table gives the statistical values associated with the symbols used in the main table. The number of data points dropped refers to the number of outliers with residuals more than 2.5 SD from the mean were removed after the initial model was built.
